# Cortisol Testing to Diagnose Adrenal Insufficiency Following Adrenalectomy for Mild Autonomous Cortisol Secretion

**DOI:** 10.1210/clinem/dgaf515

**Published:** 2026-02-20

**Authors:** Oksana Hamidi, Bahaa Salama, Tracy Wang, James W. Findling, Catherine D. Zhang, Ty Carroll, Sophie Dream, Alaa Sada, Vania Balderrama-Brondani, Prerna Dogra, Alan Dackiw, Sasan Mirfakhraee, Ankeeta Mehta, Sarah Oltmann, Travis McKenzie, Trenton Foster, Irina Bancos

**Affiliations:** 1 Division of Endocrinology, University of Texas Southwestern Medical Center, Dallas, TX 75390, USA; 2 Division of Endocrinology, Diabetes, and Nutrition, Mayo Clinic, Rochester, MN 55905, USA; 3 Division of Surgical Oncology, Section of Endocrine Surgery, Medical College of Wisconsin, Milwaukee, WI 53226, USA; 4 Division of Endocrinology and Molecular Medicine, Department of Medicine, Medical College of Wisconsin, Milwaukee, WI 53226, USA; 5 Division of Surgical Oncology, Department of Surgery, Medical College of Wisconsin, Milwaukee, WI 53226, USA; 6 Department of Surgery, Division of Endocrine Surgery, University of Pittsburgh, Pittsburgh, PA 15260, USA; 7 Department of Endocrine Neoplasia and Hormonal Disorders, University of Texas MD Anderson Cancer Center, Houston, TX 77030, USA; 8 Department of Head and Neck-Endocrine Oncology, Moffitt Cancer Center, Tampa, FL 33612, USA; 9 Department of Surgery, University of Texas Southwestern Medical Center, Dallas, TX 75390, USA; 10 Division of Endocrine Surgery, Mayo Clinic, Rochester, MN 55905, USA

**Keywords:** basal cortisol, cosyntropin stimulation test, adrenal insufficiency, hypercortisolism, adrenalectomy, adrenal adenoma

## Abstract

**Context:**

Patients with mild autonomous cortisol secretion (MACS) can develop adrenal insufficiency after unilateral adrenalectomy.

**Objective:**

This work aimed to determine the prevalence, duration, and predictors of adrenal insufficiency, and compare postoperative basal cortisol and cosyntropin stimulation test (CST).

**Methods:**

In this multicenter retrospective study of patients with MACS, adrenal insufficiency was diagnosed when postoperative basal cortisol was less than 10 μg/dL and/or CST < 18 μg/dL. The results were concordant when both tests met cutoffs. Biochemical (BSS) and clinical severity scores (CSS) were calculated.

**Results:**

Among 281 patients with MACS (80% women), postoperative adrenal insufficiency was diagnosed in 153 (54.5%) patients. Adrenal insufficiency inversely correlated with age (odds ratio [OR] 0.67 per 10 years; 95% CI, 0.53–0.84). Low basal cortisol and CST were associated with younger age (OR 0.64 per 10 years for both) and higher BSS (OR 1.17 and 1.22, respectively). Basal cortisol and CST were discordant in 22% of patients, and the discordance rate was more common in patients with bilateral nodules (32% vs 19%; *P* < .001). The median time to adrenal insufficiency recovery was 3.9 months (IQR, 3–5.9 months), with longer duration associated with higher BSS and CSS: 3 months for mild, 4 to 5 months for moderate, and 12 to 14 months for severe BSS and CSS.

**Conclusion:**

Approximately half of patients with MACS developed postoperative adrenal insufficiency, with 50% recovering by 3.9 months after surgery. BSS correlated with basal cortisol, CST, and duration of adrenal insufficiency. As discordant results were noted in 22%, performing both basal cortisol and CST postoperatively should be considered.

Mild autonomous cortisol secretion (MACS) is the most common hormonal abnormality in patients with adrenal adenoma, diagnosed in approximately 50% of cases (1–3). MACS is identified by serum cortisol exceeding 1.8 μg/dL following an overnight 1-mg dexamethasone suppression test (DST), with a clinical profile that lacks overt features of Cushing syndrome and demonstrates adrenocorticotropin (ACTH) independence (2). Adrenalectomy in patients with MACS improves cortisol-attributable comorbidities and clinical outcomes, though it carries a risk for adrenal insufficiency after removal of the adrenal source of cortisol (2, 4).

Patients undergoing unilateral adrenalectomy for MACS often require glucocorticoid supplementation for postoperative adrenal insufficiency due to long-standing chronic suppression of hypothalamic-pituitary-adrenal (HPA) axis and atrophy of the contralateral adrenal gland that develops during active hypercortisolism. At present, there is no consensus on the optimal postoperative evaluation for adrenal insufficiency. European Society of Endocrinology clinical practice guidelines recommend perioperative glucocorticoid treatment at surgical stress doses in all patients undergoing surgery for MACS (2). In contrast, American Association of Endocrine Surgeons guidelines recommend that in patients with MACS, postoperative day 1 (POD1) morning basal cortisol or cosyntropin stimulation testing (CST) could be used to determine the need for glucocorticoid replacement therapy (5). Glucocorticoid treatment is required until adrenal function recovery is documented. The prevalence of adrenal insufficiency following adrenalectomy for MACS varies widely across studies, ranging between 0% and 50%, with pooled data suggesting rates as high as 65.3% (6). Such variation reflects differences in each study criteria for diagnosis of MACS and adrenal insufficiency, including DST protocols and cortisol cutoffs.

Higher DST cortisol, lower plasma ACTH, and serum dehydroepiandrosterone sulfate (DHEA-S) are associated with the development of postoperative adrenal insufficiency (6–8). Similarly, patients with a combination of elevated DST cortisol, urinary free cortisol, midnight serum cortisol, or ACTH less than 10 pg/mL face the highest risk of developing postsurgical adrenal insufficiency (9). Additional factors, such as tumor size and cortisol-attributable comorbidities including diabetes, hypertension, and obesity, have been associated with adrenal insufficiency (10). However, identifying at-risk patients and diagnosing adrenal insufficiency remains challenging, leading to potential overtreatment with glucocorticoids.

The utility of CST for detecting adrenal insufficiency in patients with MACS remains largely unexplored, as does any comparative analysis of basal cortisol and CST responses in relation to adenoma laterality. Furthermore, the duration of glucocorticoid replacement therapy and HPA axis recovery is variable. A systematic review and meta-analysis of studies on patients with MACS indicated a mean HPA axis recovery period of 6.5 months, though the timeframe ranged from 1 to 144 months across studies (6). In a smaller cohort, the median glucocorticoid replacement duration was 55 days (interquartile range [IQR], 36–133 days), but small sample size tempers confidence in these findings (11).

In this multicenter cohort study, we aimed 1) to determine the prevalence, duration, and factors associated with the development of adrenal insufficiency after unilateral adrenalectomy for MACS, and 2) to assess the utility of CST and compare to basal cortisol to diagnose postoperative adrenal insufficiency in patients with unilateral and bilateral adrenal nodules.

## Materials and Methods

### Study Design and Participants

We performed a multicenter retrospective American Australian Asian Adrenal Alliance (A5) study on adult patients (age ≥18 years) diagnosed with MACS between January 1, 2013, and August 31, 2024. Five centers contributed data to the project: Medical College of Wisconsin (n = 137), Mayo Clinic (n = 64), University of Texas Southwestern Medical Center (n = 57), The University of Texas MD Anderson (n = 12), and University of Pittsburgh Medical Center (n = 11) (Supplemental Fig. S1) (12). This retrospective study was approved by the institutional review board of University of Texas Southwestern Medical Center, Mayo Clinic, and each participating institution, with a waiver of informed consent. Only patients who had previously provided written authorization for research use of their health information were included. All research complied with the Declaration of Helsinki. Electronic medical records and corresponding databases of patients with MACS evaluated during the study period were reviewed for confirmation of biochemical and histologic diagnosis, radiologic evaluation, management, and follow-up data. Patients with biochemically, radiologically, and/or histologically (when available) confirmed pheochromocytoma, adrenocortical carcinoma, myelolipoma (macroscopic fat), adrenal cyst, or other noncortical adrenal tumors were excluded from this study. Additionally, we excluded patients with overt Cushing syndrome, history of bilateral adrenalectomy or partial adrenalectomy of the contralateral adrenal gland, patients who did not undergo DST prior to surgery, or patients treated with perioperative or postoperative glucocorticoids empirically without formal biochemical assessment of adrenal function with basal cortisol and CST.

### Imaging Assessment

Imaging characteristics were assessed by reviewing images and imaging reports. When available, adenoma size was quantified from computed tomography (CT) and/or magnetic resonance imaging acquired most recently prior to surgery. All patients had histologically confirmed adrenal cortical adenoma with biochemical evidence of MACS. A CT or magnetic resonance imaging linear measurement tool was used to determine the largest diameter of the adrenal mass in the axial plane. To determine the CT attenuation values, an oval region-of-interest cursor was used. The region of interest covered two-thirds to three-quarters of the adrenal mass. The mass boundary, calcifications, and areas of heterogeneity were avoided.

### Hormonal Assessments

MACS was defined as failure to suppress cortisol below 1.8 μg/dL after overnight 1-mg or 8-mg dexamethasone administration in the absence of overt clinical features of hypercortisolism. For those who underwent the 8-mg DST (n = 37), we applied a more stringent threshold of cortisol less than 1.0 μg/dL to exclude autonomous cortisol secretion, and none suppressed below 1.0 μg/dL. Based on their initial 1-mg DST results and overall clinical context, these patients were classified as having MACS and included in the final analysis. At the time of surgery, the patients were not routinely given perioperative or postoperative glucocorticoids per institutional protocol. Perioperative administration of dexamethasone, including for anesthesia induction, prophylaxis of postoperative nausea, or other indications, was excluded to the greatest extent possible through detailed review of patient records. However, undocumented administration cannot be definitively excluded. The morning of POD1, serum cortisol was collected before (basal) and then 30 and 60 minutes after administration of cosyntropin 250 mcg intramuscularly or subcutaneously. Adrenal insufficiency status was assessed in 3 ways: (1) based on each center’s clinical diagnosis that incorporated basal cortisol levels, 60-minute post-ACTH stimulation cortisol levels, and clinical symptoms; (2) using basal cortisol thresholds (<10 vs ≥10 μg/dL); and (3) using stimulated cortisol thresholds (<18 vs ≥18 μg/dL). When basal cortisol and CST were discordant, the diagnosis of adrenal insufficiency was made based on the clinical decision of the treating provider. Due to the multicenter nature of our study and the broad time span over which data were collected (2013–2024), multiple cortisol assays and local protocols were used. Accordingly, we relied on each participating center’s definition of adrenal insufficiency, which did not always involve a uniform cortisol cutoff. All patients diagnosed with adrenal insufficiency were treated with hydrocortisone or prednisone per local protocol. HPA axis recovery was reassessed by repeating basal cortisol every 4 to 8 weeks postoperatively. HPA axis recovery was defined as basal cortisol greater than or equal to 10 μg/dL (usually measured 8–9 AM, after holding glucocorticoid dose for at least 24 hours) and no symptoms of persistent adrenal insufficiency or glucocorticoid withdrawal syndrome. Adrenal insufficiency duration was recorded. All biochemical testing was performed per local standards of each collaborating center.

### Classification of Mild Autonomous Cortisol Secretion

To characterize the extent of MACS, we used previously developed clinical and biochemical scoring systems (13) to grade severity based on Endocrine Society guidelines on the diagnosis of Cushing syndrome (14), European Society of Endocrinology clinical guidelines on adrenal incidentaloma (15), systematic review on MACS (16), and expert opinion (Supplementary Tables S1 and S2) (12).

### Biochemical Disease Severity

Biochemical severity assessment included evaluation of hormonal abnormalities diagnostic of hypercortisolism (24-hour urinary free cortisol, DST cortisol, and late-night salivary cortisol), and degree of ACTH independence in patients with MACS (basal ACTH and age-/sex-adjusted cutoff for DHEA-S). As patients may not have completed all available diagnostic tests, we used the sum of the 2 most abnormal tests to calculate the final biochemical severity score (BSS) (13, 15–17) (see Supplementary Table S1) (12).

### Clinical Disease Severity

Clinical disease severity was assessed by evaluating for presence of cortisol-attributable metabolic and physical findings (see Supplementary Table S2) (12). Metabolic abnormalities assessed included hypertension, prediabetes or type 2 diabetes mellitus, decreased bone density (osteopenia, osteoporosis with or without fragility fractures), and venous thromboembolic events. Physical examination findings assessed included central obesity, presence of supraclavicular and/or dorsocervical fat pads, rounding of the face with or without plethora, skin changes (acne, violaceous striae, thinning, and/or bruising of the skin), and subjective complaint of proximal muscle weakness. The final CSS was calculated by adding all metabolic and clinical examination findings for each patient prior to surgical treatment for hypercortisolism (13, 15–17).

### Statistics

Descriptive statistics were used to provide summary of the data. All continuous data were summarized as median (minimum to maximum range, or IQR), while categorical data were summarized as absolute and relative frequencies (percentages). Associations between variables were assessed using the *t* test and analysis of variance for continuous variables and chi-square test for categorical variables. For consistency in data analysis, a cortisol cutoff of less than 18 μg/dL at 60 minutes was applied across all participants. Additional analyses were performed using baseline cortisol thresholds (<10 vs ≥10 μg/dL) and comparing data against pre-2017 and post-2017 guideline updates (Supplementary Table S3) (12). To compare medians between two independent groups, we used the nonparametric Wilcoxon rank sum test. A multivariable logistic regression model was fit using factors associated with outcomes of interest. The variables assessed in the multivariable logistic regression model were prespecified and selected based on available literature. Primary and senior authors prespecified the variables based on prior literature and their experience. All tests were 2-sided, and a *P* value less than .05 was considered statistically significant. All statistical analyses were conducted using JMP version Pro 18 (SAS Institute).

## Results

### Patients With Mild Autonomous Cortisol Secretion and Unilateral vs Bilateral Adrenal Nodules

**Figure 1.**
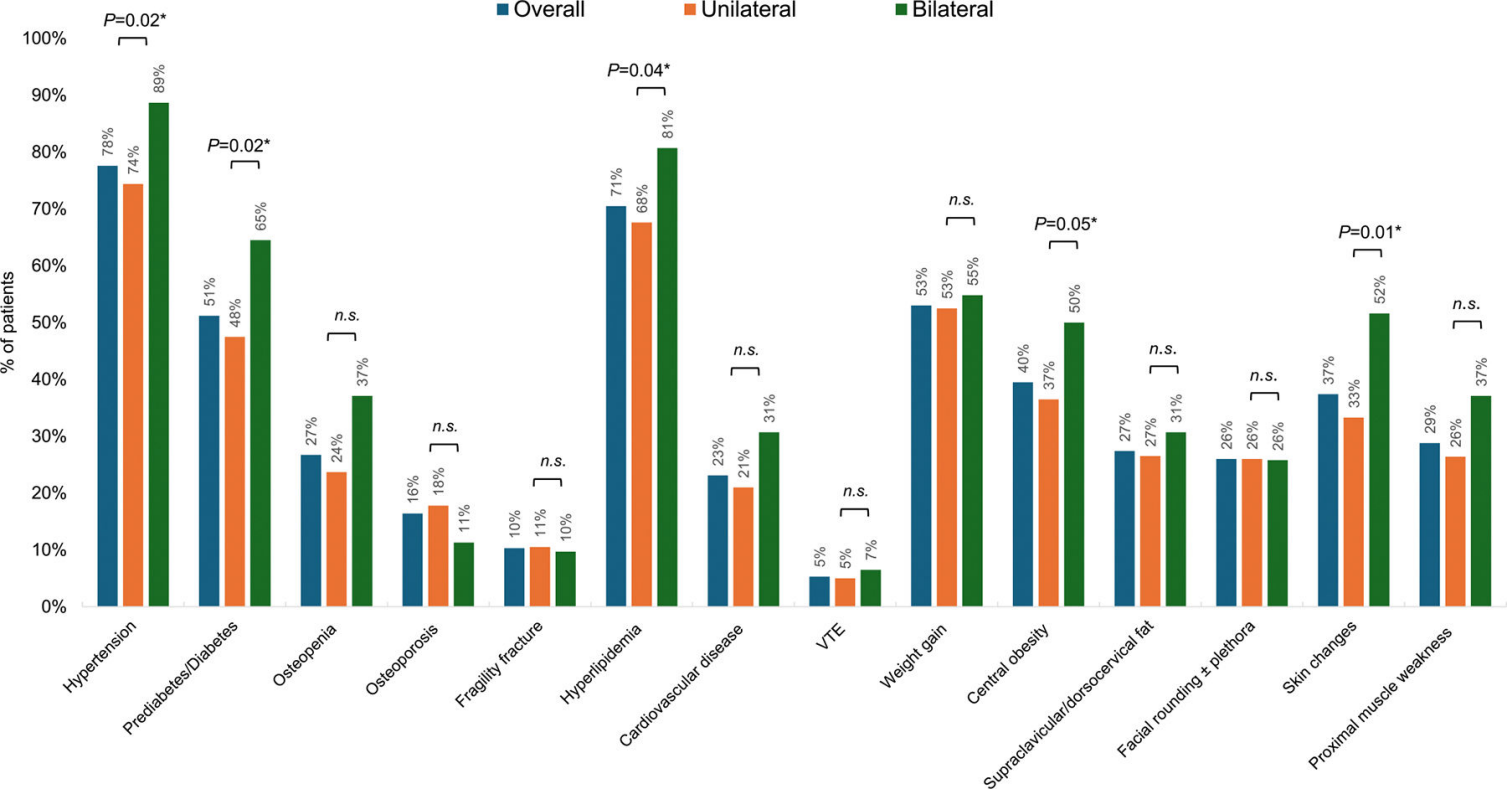
Prevalence of cardiometabolic comorbidities in patients with mild autonomous cortisol secretion (MACS), stratified by laterality of adrenal nodules. This figure compares the prevalence of key cardiometabolic comorbidities (ie, hypertension, impaired glucose metabolism, hyperlipidemia, and low bone density) and clinical signs and symptoms of hypercortisolism (ie, central obesity, skin changes, and proximal muscle weakness) between patients with unilateral adrenal nodules and those with bilateral adrenal nodules. Comorbidity definitions were based on standard clinical criteria. Differences in prevalence between the two groups were assessed using appropriate statistical tests. Statistically significant differences (*P* < .05) are indicated with an asterisk (*). n.s., not significant.

Baseline demographic, clinical, biochemical, and radiological characteristics in patients with MACS are shown in Table 1. A total of 281 patients with MACS were included in our study. Median age was 57 years (IQR, 47–64 years), and 226 (80%) were women. Mode of adenoma discovery was incidental (233, 83%), adrenal hormone excess (30, 11%), cancer surveillance (14, 5%), or genetic-based case detection/other (4, 1%). In the entire cohort, 219 (78%) patients had unilateral adenoma, whereas 62 (22%) had bilateral nodules. Compared to those with bilateral disease, patients with unilateral adenoma were younger (median age, 55 vs 58 years; *P* = .02), had smaller resected adenoma size (median, 2.9 cm vs 3.5 cm; *P* = .001), and higher unenhanced CT density (12 vs 7 Hounsfield units [HU], *P* = .014) (see Table 1). BSS was comparable among the two groups (mean, 3.9 vs 4.3; *P* = .10). In contrast, patients with bilateral nodules had higher CSS (mean, 8.1 vs 6.5; *P* = .02), suggesting higher clinical burden of dysregulated cortisol secretion in patients with bilateral nodules, compared to those with unilateral adenoma at the time of surgery (Fig. 1).

### Adrenal Insufficiency After Unilateral Adrenalectomy for Mild Autonomous Cortisol Secretion

In our cohort, 153 (54.4%) patients were diagnosed with adrenal insufficiency and treated with glucocorticoids following unilateral adrenalectomy for MACS. Patients who developed adrenal insufficiency were more likely to be women (85% vs 75%; *P* = .03), were younger (median, 54 vs 60 years; *P* < .0001), had larger resected adenoma size (median, 3.2 vs 2.7 cm; *P* = .02), higher DST cortisol (3.8 vs 3.0 μg/dL; *P* = .01), and lower baseline ACTH (6.2 vs 9.2 pg/dL; *P* = .004). Accordingly, adrenal insufficiency was associated with higher BSS (mean 4.2 vs 3.7; *P* = .04) (Table 2).

The overall prevalence of adrenal insufficiency was highest in younger patients (age <60 years), ranging from 58% to 85%, and declined steadily, with the lowest prevalence observed in those older than 60 years, ranging from 35% to 45% (Supplementary Fig. S2 (12), Supplementary Table S4) (12). When stratified by adenoma laterality, a similar age-related correlation was seen in patients with unilateral adenoma (R^2^ = 0.64), with a higher prevalence in younger patients and a gradual decline with age. In contrast, among patients with bilateral nodules (R^2^ = 0.84), the prevalence of adrenal insufficiency was 100% in those younger than 45 years and decreased to 10% to 33% in patients older than age 60 (see Supplementary Fig. S2) (12).

**Figure 2.**
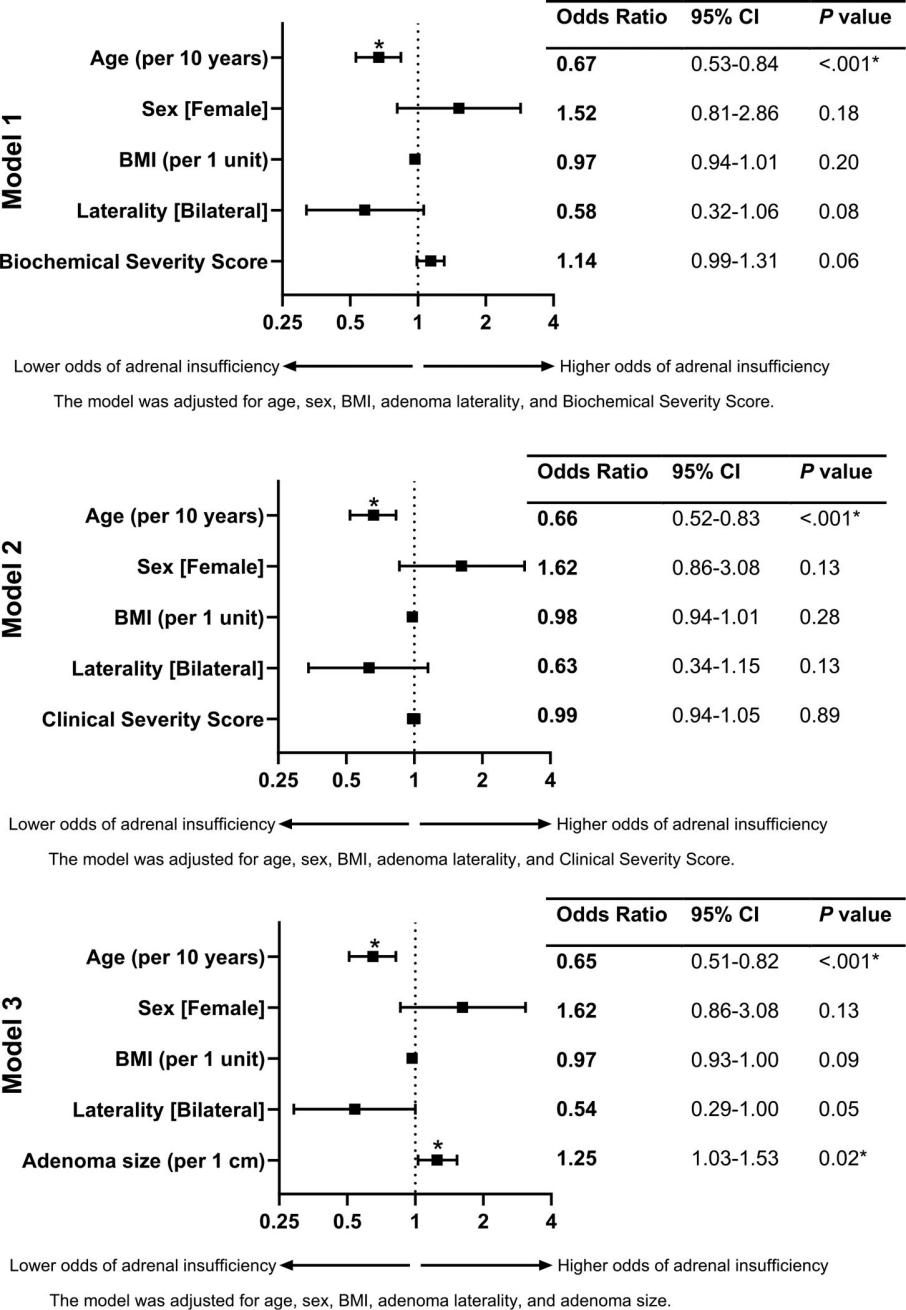
Multivariable regression analysis of risk factors for adrenal insufficiency following unilateral adrenalectomy in patients with mild autonomous cortisol secretion (MACS). Three logistic regression models were constructed to assess the association between clinical and biochemical variables and the risk of postoperative adrenal insufficiency. Model 1 included baseline demographic and clinical variables (age, sex, body mass index [BMI], adrenal nodule laterality) and a biochemical severity score for hypercortisolism. Model 2 included model 1 baseline demographic and clinical variables plus a clinical severity score for hypercortisolism. Model 3 included all baseline demographic and clinical variables in models 1 and 2 with the addition of radiological characteristics (tumor size and laterality). Results are presented as adjusted odds ratios (ORs) with 95% CIs. Statistically significant risk factors (*P* < .05) are denoted with an asterisk (*).

On univariable analysis, age (odds ratio [OR]; 95% CI, 0.08 per 10 years, 0.02–0.30), female sex (1.88, 1.04–3.45), and BSS (1.14 per 1 point, 1.01–1.3) were associated with increased risk for postoperative adrenal insufficiency. After adjusting for age, sex, body mass index (BMI), adenoma laterality, BSS, and CSS, age remained the only statistically significant factor in all models, with an OR of 0.67 (0.53–0.84 in model 1) and 0.66 (0.52–0.83 in model 2) (Fig. 2, Supplementary Table S5) (12). When further adjusting for adenoma size along with age, sex, BMI, and adenoma laterality, both age and adenoma size were associated with adrenal insufficiency. Age continued to be protective (OR 0.65 per 10 years, 0.51–0.82) (see Supplementary Table S5) (12), while larger adenomas associated with a higher risk of adrenal insufficiency (OR 1.25, 1.03–1.53) (see Fig. 2).

### Postoperative Day 1 Basal Cortisol

**Figure 3.**
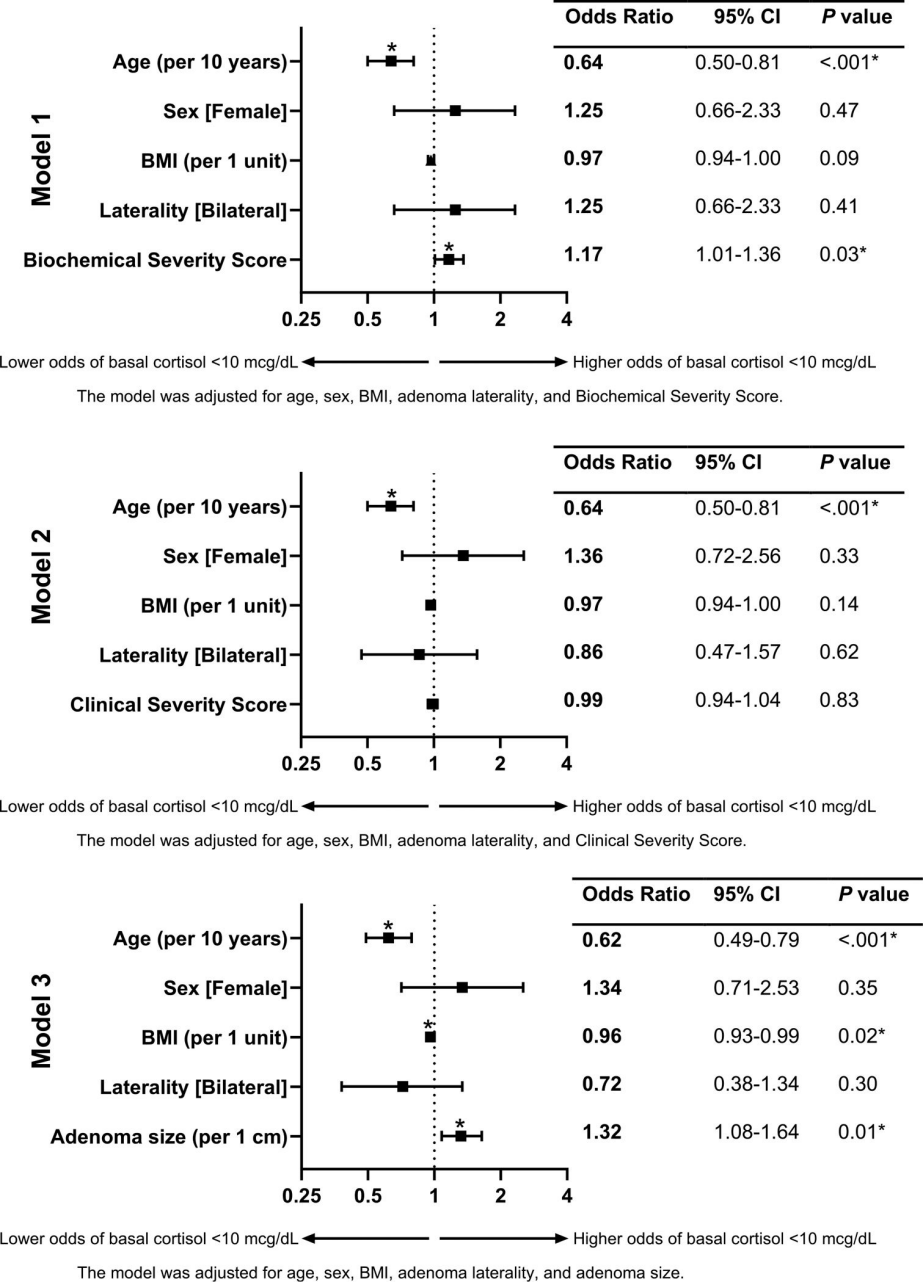
Multivariable regression analysis of postoperative basal cortisol less than 10 μg/dL. following unilateral adrenalectomy for mild autonomous cortisol secretion (MACS). The figure presents adjusted odds ratios (ORs) with 95% CIs across 3 logistic regression models, each assessing the independent association of basal cortisol less than 10 μg/dL. Model 1 is adjusted for age, sex, body mass index (BMI), adrenal nodule laterality, and a biochemical severity score. Model 2 is adjusted for age, sex, BMI, adrenal nodule laterality, and a clinical severity score. Model 3 is adjusted for age, sex, BMI, adrenal nodule laterality, and adenoma size. Statistically significant associations (*P* < .05) are indicated with an asterisk (*).

Early-morning basal cortisol was measured postoperatively for all patients. We observed that 169 (60.1%) patients had basal cortisol less than 10 μg/dL, which was associated with younger age (54 vs 60 years; *P* < .001), larger resected adenoma size (3.1 vs 2.7 cm; *P* = .02), lower baseline ACTH (6 vs 9.9 pg/dL; *P* < .0001), and higher BSS (4.2 vs 3.7; *P* = .02), compared to those with basal cortisol greater than or equal to 10 μg/dL (Table 3). Conversely, DST, DHEA-S, and CSS did not reveal an association with low basal serum cortisol (see Table 3). On multivariable analysis, factors associated with low basal cortisol level less than 10 μg/dL included younger age (OR 0.64; 95% CI, 0.50–0.81) and higher BSS (1.17; 1.01–1.36) after adjusting for sex, BMI, and adenoma laterality (Fig. 3, Supplementary Table S6) (12).

### Cosyntropin Stimulation Test

**Figure 4.**
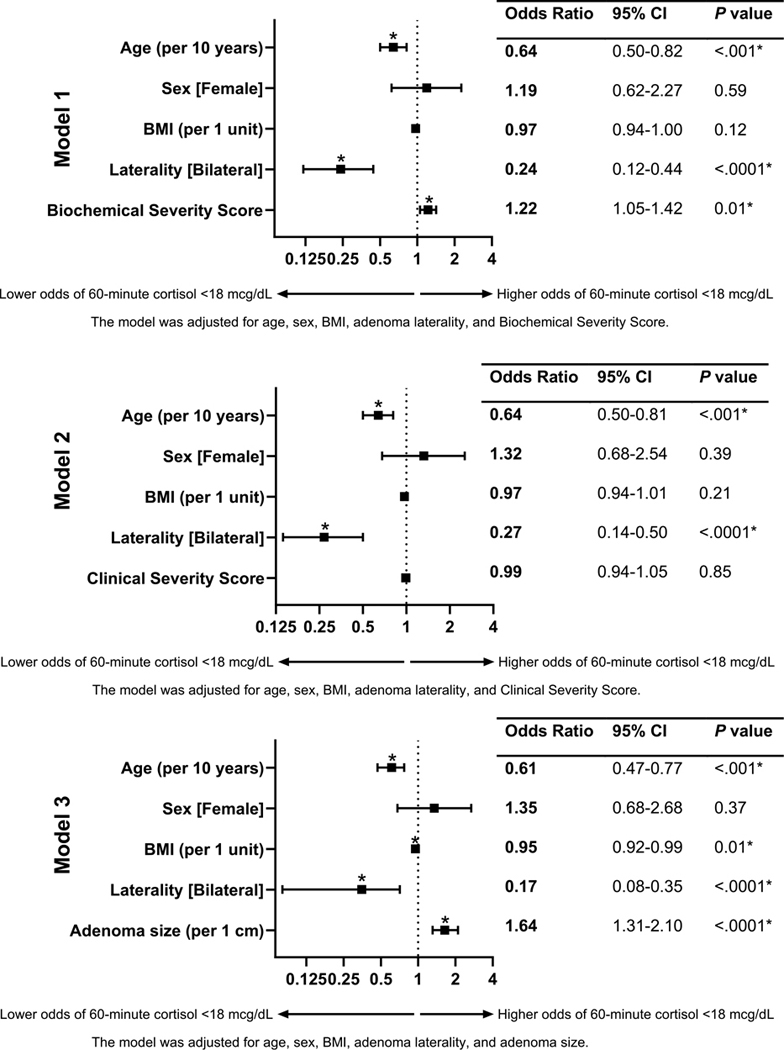
Multivariable regression analysis of postoperative peak cortisol less than 18 μg/dL on cosyntropin stimulation testing, following unilateral adrenalectomy for mild autonomous cortisol secretion (MACS). The figure presents adjusted odds ratios (ORs) with 95% CIs across 3 logistic regression models, each assessing the independent association of peak cortisol less than 18 μg/dL on postoperative cosyntropin stimulation testing. Statistically significant associations (*P* < .05) are indicated with an asterisk (*). Model 1 is adjusted for age, sex, body mass index (BMI), adrenal nodule laterality, and a biochemical severity score for hypercortisolism. Model 2 is adjusted for age, sex, BMI, adrenal nodule laterality, and a clinical severity score for hypercortisolism. Model 3 is adjusted for age, sex, BMI, adrenal nodule laterality, and adenoma size. Statistically significant associations (*P* < .05) are indicated with an asterisk (*).

The 60-minute cortisol following CST was measured postoperatively in the entire cohort. We observed that 162 (57.6%) patients had a 60-minute cortisol less than 18 μg/dL. Factors associated with a 60-minute cortisol less than 18 μg/dL included younger age, unilateral adenoma, larger resected tumor size, lower baseline ACTH and DHEA-S concentrations, higher 1-mg DST cortisol, and higher BSS (Table 4). On multivariable analysis, younger age (OR 0.64 per 10 years; 95% CI, 0.50–0.81) and higher BSS (OR 1.22 per each point; 95% CI, 1.05–1.42) were associated with a CST less than 18 μg/dL, whereas presence of bilateral adrenal nodules was associated with lower risk for low 60-minute cortisol (OR 0.24 for bilateral adenoma; 95% CI, 0.12–0.44), after adjusting for age, sex, and BMI (Fig. 4, Supplementary Table S7) (12).

**Figure 5.**
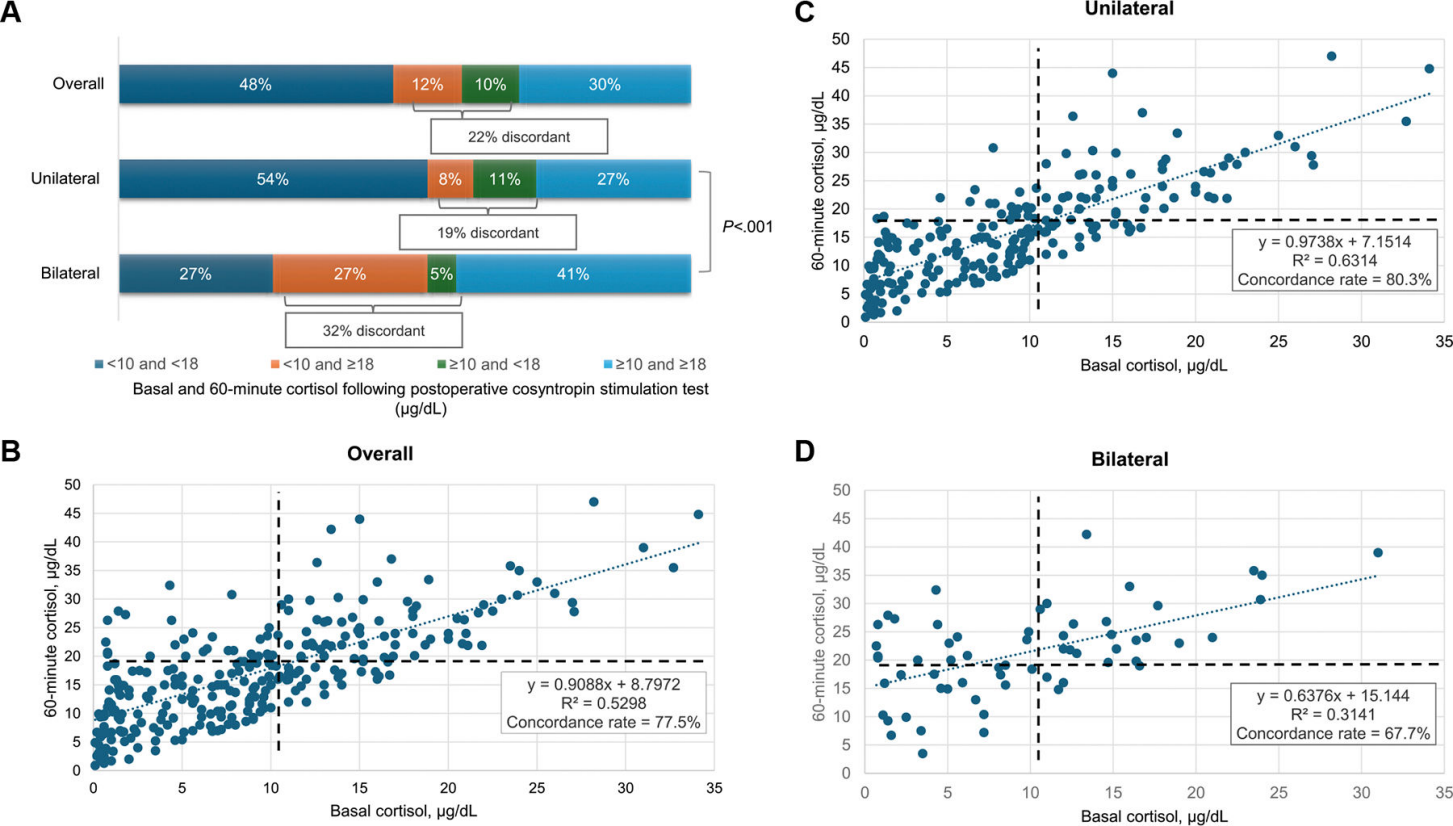
Distribution of basal and stimulated cortisol values and concordance patterns on postoperative biochemical evaluation for adrenal insufficiency following unilateral adrenalectomy for mild autonomous cortisol secretion (MACS). This figure illustrates the relationship between basal cortisol levels and peak cortisol responses following cosyntropin stimulation in patients with MACS, highlighting concordant and discordant test results across different subgroups. Panel A displays the concordance and discordance between basal cortisol less than 10 μg/dL and peak cortisol less than 18 μg/dL on cosyntropin stimulation testing. Concordant results include patients with both low basal and low stimulated cortisol values, or both above the respective thresholds. Discordant results represent mismatched basal and peak cortisol classifications. Panel B shows the distribution of basal and 60-minute stimulated cortisol values for the entire cohort, including the calculated coefficient of determination (R^2^) and the overall concordance rate between basal and peak cortisol classifications. Panel C presents the basal and stimulated cortisol distributions specifically for patients with unilateral adrenal nodules, along with R^2^ and concordance rate. Panel D presents the same distributions for patients with bilateral adrenal nodules, also including R^2^ and concordance rate. Dashed lines indicate the threshold values for basal cortisol (<10 μg/dL) and peak cortisol (<18 μg/dL).

The overall concordance rate between basal and 60-minute cortisol was 78% for the diagnosis of adrenal insufficiency, with lower concordance in patients with bilateral nodules than those with unilateral adenoma (68% vs 81% concordance). Patients with bilateral nodules were more likely to have discordant results (32% vs 19%; *P* < .001), with more cases of basal cortisol less than 10 μg/dL but 60-minute cortisol of 18 μg/dL or greater (27% with bilateral vs 8% with unilateral) (Fig. 5).

### Adrenal Insufficiency Recovery

**Figure 6.**
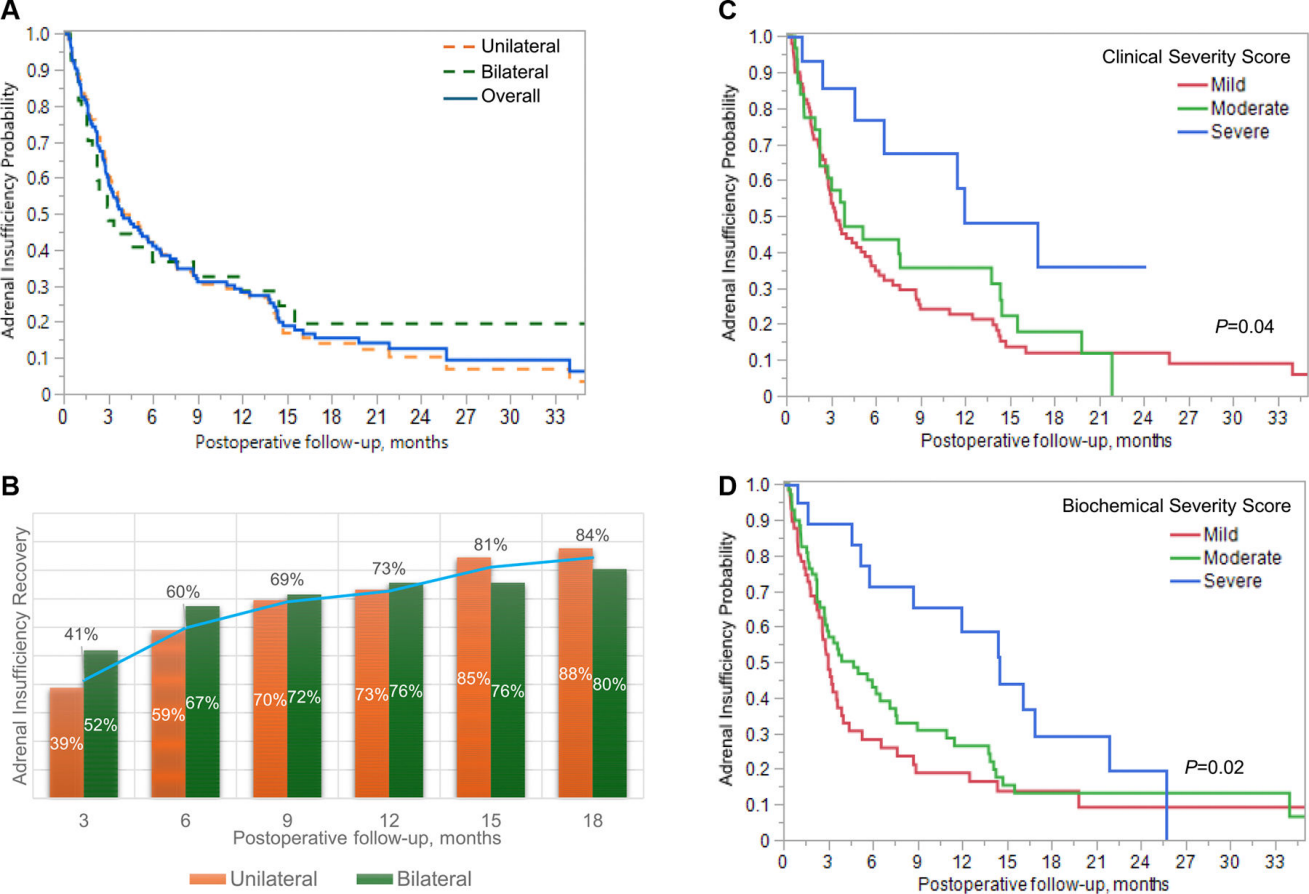
Recovery of adrenal insufficiency over time following unilateral adrenalectomy in patients with mild autonomous cortisol secretion (MACS). This figure illustrates the relationship between basal cortisol levels and peak cortisol responses following cosyntropin stimulation in patients with MACS, highlighting concordant and discordant test results across different subgroups. Panel A displays the concordance and discordance between basal cortisol less than 10 μg/dL and peak cortisol less than 18 μg/dL on cosyntropin stimulation testing. Concordant results include patients with both low basal and low stimulated cortisol values, or both above the respective thresholds. Discordant results represent mismatched basal and peak cortisol classifications. Panel B shows the distribution of basal and 60-minute stimulated cortisol values for the entire cohort, including the calculated coefficient of determination (R^2^) and the overall concordance rate between basal and peak cortisol classifications. Panel C presents the basal and stimulated cortisol distributions specifically for patients with unilateral adrenal nodules, along with R^2^ and concordance rate. Panel D presents the same distributions for patients with bilateral adrenal nodules, also including R^2^ and concordance rate. Dashed lines indicate the threshold values for basal cortisol (<10 μg/dL) and peak cortisol (<18 μg/dL).

During a median postoperative follow-up period of 15.4 months, 108 patients (70.6%) recovered from adrenal insufficiency and discontinued glucocorticoids. The median duration of adrenal insufficiency was 3.9 months (95% CI, 3.0–5.9 months) in the overall cohort, 4.0 months (3.1–6.5 months) in patients with unilateral adenoma vs 3.0 months (1.6–11.9 months) in patients with bilateral nodules (Fig. 6). Among those who developed adrenal insufficiency, 41% recovered by 3 months, 60% by 6 months, 73% by 12 months, and 84% by 18 months. The shorter median duration of adrenal insufficiency was associated with lower biochemical severity: 3 months for mild, 4.7 months for moderate, and 14.5 months for severe (*P* = .02) (Supplementary Table S8) (12). In parallel, shorter time to recovery was also associated with lower CSS: 3.3 months for mild, 3.9 months for moderate, and 11.9 months for severe (*P* = .04) (see Fig. 6).

## Discussion

Our large multicenter study showed that approximately half of patients developed postoperative adrenal insufficiency following unilateral adrenalectomy for MACS. By comparing postoperative basal cortisol and CST, we demonstrated discordant diagnosis in 22% of patients, higher in those with bilateral adrenal nodules. We further showed that BSS predicts postadrenalectomy low basal cortisol and CST, whereas both BSS and CSS predict the duration of postoperative adrenal insufficiency, with 50% of patients recovering by 4 months post surgery. These findings indicate the importance of prompt postoperative assessment of the HPA axis to accurately diagnose adrenal insufficiency and the need for appropriate monitoring of HPA axis recovery.

We found that 53.5% of patients developed postoperative adrenal insufficiency. A similar prevalence of adrenal insufficiency of 50% to 60% was reported in prior studies, though the definition of MACS and the approach to diagnosing adrenal insufficiency varied (6, 18, 19). Identifying patients at highest risk for adrenal insufficiency following unilateral adrenalectomy for MACS remains challenging. We observed that younger patients were more likely to develop postoperative adrenal insufficiency. Namely, the overall rate of adrenal insufficiency was 2- to 3-fold higher in patients younger than 60 years, compared to those older than 60 years. Younger patients were more commonly women and had lower preoperative ACTH and higher 1-mg DST, though association with age remained statistically significant in multivariable analysis. The age-related risk of adrenal insufficiency has not yet been demonstrated in prior studies, which warrants further confirmation.

Cortisol-attributable comorbidities at baseline (as measured by CSS) were not associated with adrenal insufficiency. However, we determined that BSS was associated with postoperative adrenal insufficiency, defined either based on the abnormal basal cortisol or abnormal post-CST cortisol. BSS is calculated using the two most abnormal biochemical test results, as previously reported (13, 20), which inherently reflects the degree of MACS. We found that for each additional BSS point increase, the risk for postoperative low basal cortisol and/or low CST was approximately 20% higher, independent of age, sex, BMI, and adenoma laterality.

Bilateral adrenal nodules are reported in 15% to 20% of incidentally discovered adrenal masses (1, 21, 22), with up to 26% in patients with MACS as shown in our prior study (23). Yet, whether the risk for adrenal insufficiency following unilateral adrenalectomy differs based on laterality has not been systematically investigated. In our study, prevalence of adrenal insufficiency was lower in patients with bilateral nodules (44%), as opposed to 58% in patients with unilateral adenoma. Lower prevalence of adrenal insufficiency in patients with bilateral nodules is potentially due to a lesser degree of HPA axis suppression and/or persistent autonomous cortisol secretion from the contralateral adrenal gland. Such patients may need to be reassessed with 1-mg DST postoperatively (once off glucocorticoids) to confirm biochemical remission of MACS.

Although there is no consensus on the optimal assessment for adrenal insufficiency after unilateral adrenalectomy for MACS, adrenal function can be assessed by either basal cortisol or CST on POD1. Scarce data are available comparing POD1 basal cortisol and CST in this clinical setting. Implementing postoperative biochemical testing can identify patients with adrenal insufficiency, guide glucocorticoid treatment, and obviate unnecessary glucocorticoids in half of patients with MACS (18, 19). Prior studies showed that a basal cortisol threshold less than 10 μg/dL on POD1 can be used to assess adrenal function, which have been reported to selectively limit postoperative glucocorticoid replacement to approximately 50% of patients with MACS (18–20). In a prior study, POD1 CST was more discerning than POD1 basal cortisol in identifying patients with adrenal insufficiency after unilateral adrenalectomy for MACS and resulted in fewer patients receiving glucocorticoid replacement compared with the use of a basal cortisol less than 10 μg/dL for diagnosing adrenal insufficiency (19). In patients with ACTH-independent hypercortisolism, postoperative CST on POD1 reveals abnormal response in 50% to 67% of cases (19, 24). However, laterality-based analysis was not performed in that study. We hypothesized that patients with bilateral adrenal nodules may have low basal cortisol but normal CST as the contralateral adrenal gland may not be atrophic. Indeed, we showed that 27% of patients with bilateral adrenal nodules had discordant basal and post-CST cortisol. In these cases, CST may not be a reliable assessment, as with exogenous supraphysiologic synthetic ACTH, these patients may mount an appropriate cortisol response. In contrast, patients with unilateral adenoma with a basal cortisol greater than 10 μg/dL may have a suboptimal peak cortisol post CST, potentially reflecting a maximal capacity for cortisol secretion at the time of basal assessment. In these patients, representing 11% of our cohort, performing CST could provide additional diagnostic value.

We have observed a median duration of adrenal insufficiency of 3.9 months. Both preoperative BSS and CSS were associated with the duration of adrenal insufficiency. Patients with severe BSS had the longest duration of adrenal insufficiency lasting more than 1 year, compared to 3 to 4 months for patients with mild-moderate BSS. Similar findings were reported in another study that included 30 patients with MACS, in which the duration of glucocorticoid therapy also directly correlated with BSS (20). Similarly, patients with severe CSS had a longer duration of adrenal insufficiency (11.9 months), as opposed to only 3 to 4 months in those with mild or moderate CSS. BSS and CSS could be used in preoperative counseling in regard to risk and duration of adrenal insufficiency.

In our multicenter cohort, at least half of patients experienced recovery of adrenal function sooner than 3 months postoperatively, especially in the setting of bilateral adrenal nodules and those with milder BSS and CSS. In a systematic review and meta-analysis, mean time to adrenal insufficiency recovery was 6.5 months, ranging widely from 1 to 144 months (6). The shorter duration of adrenal insufficiency in our study may be related to variable protocols for postoperative follow-up and reassessment of adrenal function. Since our cohort included data from 5 different centers located across the United States, we believe that the recovery of adrenal insufficiency is quicker than previously reported. Our findings emphasize the need for earlier reassessment of HPA axis to limit unnecessary glucocorticoid exposure.

Our study has several important limitations related to its retrospective design, referral bias, information bias, the lack of standardized documentation regarding the specific clinical rationale for adrenalectomy, use of nonstandardized glucocorticoid-tapering regimens, and protocols for assessing HPA axis recovery. Moreover, we included only patients who underwent basal cortisol and CST testing on POD1, eliminating those who received empiric glucocorticoid treatment. Another potentially confounding factor is the use of perioperative dexamethasone ordered by the anesthesiologist without approval or recognition by the surgical team. In our centers, we take a proactive role in communicating with anesthesiology to avoid any perioperative dexamethasone or other glucocorticoids during unilateral adrenalectomy for MACS. Nonetheless, administration of perioperative glucocorticoids might be an important confounder when interpreting postoperative basal cortisol and DST in other centers.

Additionally, in the setting of discrepant basal cortisol and CST results, the decision whether to initiate glucocorticoid treatment was made by treating physicians, considering clinical context and individual preference. While some opted to initiate a glucocorticoid regimen based on low basal cortisol (even if CST was normal), others opted to rely on CST. In all cases, patient symptoms were considered to aid with the decision-making process when basal cortisol and CST were discordant. It is also important to consider the effect of different cutoffs of basal cortisol and CST for the diagnosis of adrenal insufficiency. We opted for a 60-minute cortisol cutoff of 18 μg/dL, in accordance with the Endocrine Society and joint European Society of Endocrinology and Endocrine Society clinical guidelines (25, 26). We acknowledge that using this cutoff, as opposed to the proposed lower CST cortisol cutoffs (27), may have resulted in overdiagnosis of adrenal insufficiency. However, missing adrenal insufficiency diagnosis can lead to multiple symptoms, poor quality of life, and adrenal crisis. In general, the HPA axis was retested once the patient was weaned to a physiologic dose of glucocorticoids, which was usually accomplished in 4 to 8 weeks postoperatively. This is highly variable and dependent on multiple patient-specific factors, such as the severity of hypercortisolism, comorbid conditions, glucocorticoid withdrawal symptoms and tolerance of taper, patient factors related to the geographical proximity to the treating center and/or laboratory facilities, and variability of follow-up. These factors could have influenced the interpretation of postoperative adrenal insufficiency duration in our cohort.

As demonstrated in our study, the frequency of adrenalectomies for MACS has significantly increased over the past decade across the centers involved. This trend can be explained by greater recognition of negative effects of MACS on cardiometabolic health and evident improvement in the outcomes following adrenalectomy, compared to conservative treatment (28). The development of postoperative adrenal crisis or readmission is rare in patients with MACS (18, 29). Only half of patients with MACS develop adrenal insufficiency postoperatively, and potentially even fewer patients with bilateral adrenal nodules (treated with unilateral adrenalectomy). Routine perioperative glucocorticoid use after adrenalectomy for MACS remains debatable. It may be appropriate in centers where timely postoperative adrenal function testing is limited, prioritizing patient safety by reducing the risk of adrenal crisis. However, whenever possible, we advocate individualized evaluation of adrenal function to guide glucocorticoid therapy, minimizing unnecessary steroid exposure and its associated risks.

In conclusion, we found that adrenal insufficiency developed in approximately half of patients following unilateral adrenalectomy for MACS, with higher prevalence in younger age groups. Higher BSS was associated with low basal cortisol, abnormal CST, and longer duration of adrenal insufficiency. Basal cortisol and CST results were concordant in only 78% of patients, with a particularly high discordance rate (32%) in patients with bilateral adrenal nodules. The additive value of performing CST in addition to POD1 basal cortisol should be considered in patients with MACS treated with unilateral adrenalectomy, when feasible. Postoperative biochemical testing can identify patients with adrenal insufficiency, guide glucocorticoid treatment, and obviate unnecessary glucocorticoids.

## Supplementary Material

Supplemental Figure 1

Supplemental Figure 2

Supplemental Tables

## Figures and Tables

**Table 1.** Baseline characteristics in patients with mild autonomous cortisol secretion

Variable	Total cohort (N = 281)	Unilateral n = 219 (78%)	Bilateral n = 62 (22%)	*P*

Sex, female, n (%)	226 (80%)	177 (80%)	49 (79%)	.72
Age, median (IQR), y	57 (47 to 64)	55 (45 to 64)	58 (51 to 65)	.02^a^
BMI, median (IQR)	32.6 (27.0 to 37.7)	32.6 (27.0 to 39.2)	32.9 (26.5 to 35.9)	.32
Time from adenoma diagnosis to adrenalectomy, median (IQR), mo	12.7 (4.9 to 46.3)	11.7 (4.7 to 46.0)	16.5 (6.8 to 60.9)	.28
Resected adenoma size, median (IQR), cm	3.0 (2.3 to 3.9)	2.9 (2.2 to 3.7)	3.5 (2.7 to 4.5)	.001^a^
Adenoma density, median (IQR), HU	10 (0 to 25)	12 (0 to 26)	7 (−6 to 13)	.014^a^
1-mg DST cortisol, μg/dL	3.3 (2.5 to 6)	3.2 (2.5 to 5.6)	3.9 (2.7 to 6.7)	.07
UFC, median (IQR), μg/24 h	30.85 (15.0 to 59.4)	27 (15 to 56.9)	39 (15.4 to 69.0)	.36
UFC <45 μg/24 h, n (%)	105/174 (60%)			
LNCS, median (IQR), ng/dL	109.7 (66.6 to 208.9)	113 (63.4 to 216.3)	101.5 (70.6 to 200.4)	.78
ACTH, median (IQR), pg/dL	7.0 (5 to 13)	4.6 (5 to 14)	6.1 (5 to 10)	.09
DHEA-S, median (IQR), μg/dL	37.1 (19 to 67)	36 (19 to 67.8)	39.6 (22.7 to 65.3)	.94
Confirmatory DST, n (%)	82 (29%)	3.4 (2.5–6.3)	3.3 (2.3–5.7)	.92
DST cortisol, median (IQR), μg/dL	3.4 (2.5 to 6.1)			
Primary aldosteronism cosecretion	30/228 (13%)	24 (13%)	6 (12%)	.83
Biochemical severity score, mean (SD)	4.0 (1.8)	3.9 (1.8)	4.3 (1.7)	.10
Clinical severity score, mean (SD)	6.9 (5.0)	6.5 (5.0)	8.1 (5.1)	.02^a^

Abbreviations: ACTH, adrenocorticotropin; BMI, body mass index, DHEA-S, dehydroepiandrosterone sulfate; DST, dexamethasone suppression test; IQR, interquartile range; LNSC, late-night salivary cortisol; UFC, urinary free cortisol.

a Statistically significant association (*P* < .05).

**Table 2.** Clinical and biochemical characteristics in patients with and without adrenal insufficiency following unilateral adrenalectomy for mild autonomous cortisol secretion

Variable	Adrenal insufficiency n = 153 (54.5%)	No adrenal insufficiency n = 128 (45.5%)	*P*

Sex, female, n (%)	130 (85%)	96 (75%)	.03^a^
Age, median (IQR), y	54 (44–64.5)	60 (51–65.8)	<.0001^a^
BMI, median (IQR)	32.3 (26.9–37.6)	33.2 (27.1–38.5)	.53
Laterality			.05^a^
Unilateral	126 (82.4%)	93 (72.7%)	
Bilateral	27 (17.7%)	35 (27.3%)	
Time from adenoma diagnosis to adrenalectomy, median (IQR), mo	11.4 (4.7–58.6)	15.3 (5.6–42.4)	.84
Resected adenoma size, median (IQR), cm	3.2 (2.5–4.0)	2.7 (2.0–3.7)	.02^a^
Resected adenoma density, median (IQR), HU	13 (0.8–25)	8.8 (0–21)	.06
1-mg DST cortisol, median (IQR), μg/dL	3.8 (2.5–6.9)	3.0 (2.5–4.5)	.01^a^
ACTH, median (IQR), pg/dL	6.2 (5–11.2)	9.2 (5–14.1)	.004^a^
DHEA-S, median (IQR), μg/dL	35 (18–70)	38 (24–65)	.46
LNCS, median (IQR), ng/dL	112.2 (65.8–233.4)	108.9 (66.1–200.5)	.50
UFC, median (IQR), μg/24 h	31.6 (16.9–66.0)	27.9 (14.4–54.6)	.38
Aldosterone cosecretion	14 (11.0%)	16 (15.8%)	.29
Biochemical severity score, mean (SD)	4.2 (1.9)	3.7 (1.6)	.04^a^
Clinical severity score, mean (SD)	6.8 (5.1)	6.9 (4.9)	.83

Abbreviations: ACTH, adrenocorticotropin; BMI, body mass index, DHEA-S, dehydroepiandrosterone sulfate; DST, dexamethasone suppression test; IQR, interquartile range; LNSC, late-night salivary cortisol; UFC, urinary free cortisol.

a Statistically significant association (*P* < .05).

**Table 3.** Baseline characteristics and hormonal profiles of patients stratified by basal cortisol concentrations (<10 μg/dL vs >10 μg/dL)

Variable	Basal cortisol <10 μg/dL n = 169 (60.1%)	Basal cortisol ≥10 μg/dL n = 112 (39.9%)	*P*

Women, n (%)	141 (83.4%)	85 (75.9%)	.11
Age, median (IQR), y	54 (44–62)	60 (52–66)	<.001^a^
BMI, median (IQR)	32.4 (26.8–37.5)	33.1 (27.2–38.4)	.41
Mode of discovery, n (%)			
Incidental	141 (83.4%)	92 (82.1%)	.77
Laterality			.33
Unilateral	135 (79.9%)	84 (75.0%)	
Bilateral	34 (20.1%)	28 (25.0%)	
Time from adenoma diagnosis to adrenalectomy, median (IQR), mo	11.6 (4.6–46.0)	15.4 (5.5–47.6)	.71
Resected adenoma size, median (IQR), cm	3.1 (2.4–4.0)	2.7 (2.0–3.7)	.02^a^
Resected adenoma density, median (IQR), HU	12 (0–25)	9 (0–22.7)	.20
ACTH, median (IQR), pg/dL	6 (5–10.9)	9.9 (5.6–14.4)	<.0001^a^
DHEA-S, median (IQR), μg/dL	37.1 (19–70)	37.5 (20.7–64.7)	.95
1-mg DST cortisol, median (IQR), μg/dL	3.4 (2.5–6.6)	3.1 (2.5–5.1)	.24
LNCS, median (IQR), ng/dL	115.7 (69.5–218.5)	100.0 (57.9–203.1)	.26
UFC in μg/24 h, median (IQR)	31.4 (17.1–63.7)	26.0 (14.3–54.6)	.25
Biochemical severity score, mean (SD)	4.2 (1.9)	3.7 (1.6)	.02^a^
Clinical severity score, mean (SD)	6.8 (5.2)	7.0 (4.8)	.71

Abbreviations: ACTH, adrenocorticotropin; BMI, body mass index, DHEA-S, dehydroepiandrosterone sulfate; DST, dexamethasone suppression test; IQR, interquartile range; LNSC, late-night salivary cortisol; UFC, urinary free cortisol.

a Statistically significant association (*P* < .05).

**Table 4.** Baseline characteristics and hormonal profiles of patients stratified by stimulated 60-minute cortisol (<18 μg/dL vs ≥18 μg/dL)

Variable	60-min cortisol <18 μg/dL n = 162 (57.6%)	60-min cortisol ≥18 μg/dL n = 119 (42.4%)	*P*

Women, n (%)	135 (83.3%)	91 (76.4%)	.15
Age, median (IQR), y	54 (44–62)	60 (51–65)	<.0001^a^
BMI, median (IQR)	32.2 (26.8–37.7)	33.1 (27.2–37.9)	.58
Laterality			<.0001^a^
Unilateral	Reference	Reference	
Bilateral	20 (12.3%)	42 (35.2%)	
Time from adenoma diagnosis to adrenalectomy, median (IQR), mo	11.4 (4.6–58.9)	15.5 (5.7–43.8)	.61
Resected adenoma size, median (IQR), cm	3.2 (2.5–4.1)	2.7 (1.9–3.6)	.001^a^
Resected adenoma density, median (IQR), HU	12 (0–25)	9.5 (0–21)	.28
ACTH, median (IQR), pg/dL	6.0 (5.0–10.9)	9.3 (5.3–14.1)	.0004^a^
DHEA-S, median (IQR), μg/dL	33.1 (17.7–67.0)	45 (26.1–69.0)	.02^a^
1-mg DST cortisol, median (IQR), μg/dL	3.7 (2.6–7.0)	3.0 (2.4–4.3)	.018^a^
LNCS, median (IQR), ng/dL	122.8 (71.9–219.7)	101.7 (63.3–200.5)	.33
UFC in μg/24 h, median (IQR)	27 (15.7–60.9)	34.1 (14.6–56.8)	.97
Biochemical severity score, mean (SD)	4.22 (1.89)	3.73 (1.65)	.02^a^
Clinical severity score, mean (SD)	6.7 (5.0)	7.1 (5.1)	.43

Abbreviations: ACTH, adrenocorticotropin; BMI, body mass index, DHEA-S, dehydroepiandrosterone sulfate; DST, dexamethasone suppression test; IQR, interquartile range; LNSC, late-night salivary cortisol; UFC, urinary free cortisol.

a Statistically significant association (*P* < .05).

## Data Availability

Restrictions apply to the availability of some or all data generated or analyzed during this study to preserve patient confidentiality or because they were used under license. The corresponding author will on request detail the restrictions and any conditions under which access to some data may be provided.

## Abbreviations:

ACTH: adrenocorticotropin
BMI: body mass index
BSS: biochemical severity score
CSS: clinical severity score
CST: cosyntropin stimulation test
CT: computed tomography
DHEA-S: dehydroepiandrosterone sulfate
DST: dexamethasone suppression test
HPA: hypothalamic-pituitary-adrenal
IQR: interquartile range
LNSC: late-night salivary cortisol
MACS: mild autonomous cortisol secretion
OR: odds ratio
POD1: postoperative day 1
UFC: urinary free cortisol
