# Supplementary material for: Cortisol Testing to Diagnose Adrenal Insufficiency Following Adrenalectomy for Mild Autonomous Cortisol Secretion
Source: J Clin Endocrinol Metab. Author manuscript; Available in PMC 2026 Apr 7. (PMC13056330; doi:10.1210/clinem/dgaf515)
Supplement: Supplemental Figure 1 [file NIHMS2153880-supplement-Supplemental_Figure_1.pdf]

Supplemental Figure 1. Temporal patterns of adrenal adenoma diagnosis and adrenalectomy.

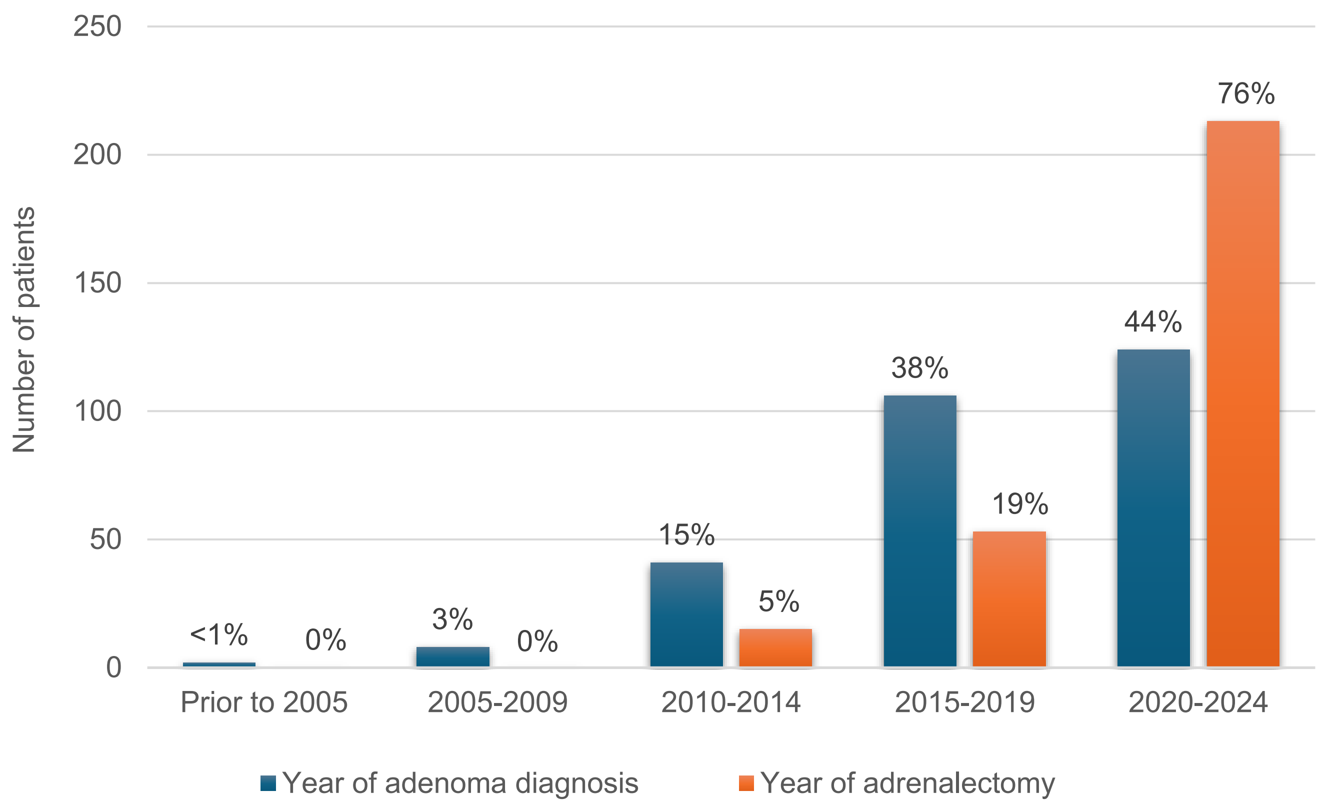

Median time from adenoma diagnosis to adrenalectomy: 12.7 months (IQR, 4.9-46.3)
