# Supplementary material for: Cortisol Testing to Diagnose Adrenal Insufficiency Following Adrenalectomy for Mild Autonomous Cortisol Secretion
Source: J Clin Endocrinol Metab. Author manuscript; Available in PMC 2026 Apr 7. (PMC13056330; doi:10.1210/clinem/dgaf515)
Supplement: Supplemental Figure 2 [file NIHMS2153880-supplement-Supplemental_Figure_2.pdf]

Supplemental Figure 1. Temporal patterns of adrenal adenoma diagnosis and adrenalectomy.

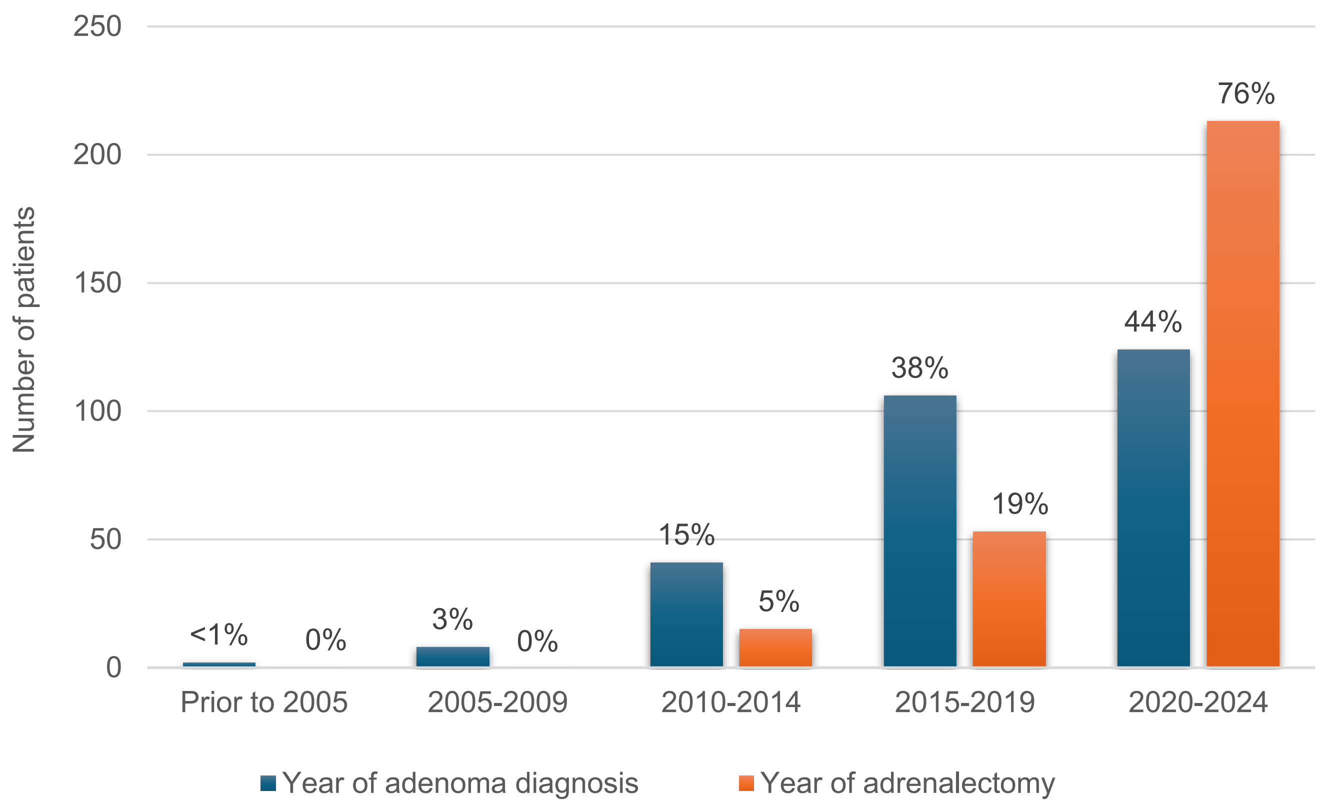

Median time from adenoma diagnosis to adrenalectomy: 12.7 months (IQR, 4.9-46.3)

Supplemental Figure 2. Age-dependent prevalence of adrenal insufficiency following unilateral adrenalectomy for MACS.

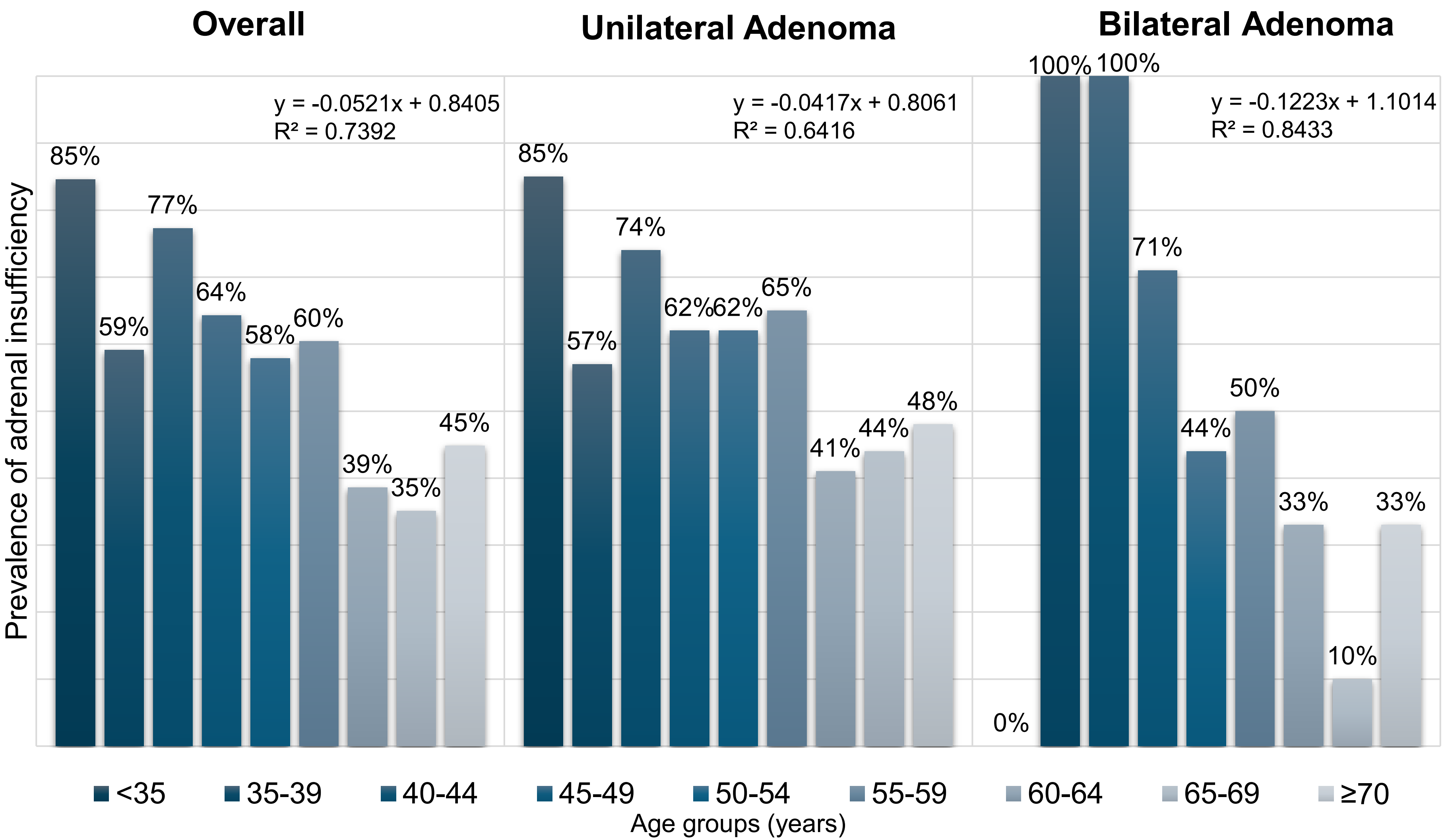

The prevalence of adrenal insufficiency was highest in younger patients (<60 years), ranging from 58-85%, and declined steadily, with the lowest prevalence observed in those older than 60 years, ranging from 35-45%. When stratified by adenoma laterality, a similar age-related correlation was seen in patients with unilateral adenoma (R^2 =0.64), with a higher prevalence in younger patients and a gradual decline with age. In contrast, among patients with bilateral adenoma (R^2 =0.84), the prevalence of adrenal insufficiency was 100% in those younger than 45 years and decreased to 10-33% in patients older than 60 years.
