# Supplementary material for: Cortisol Testing to Diagnose Adrenal Insufficiency Following Adrenalectomy for Mild Autonomous Cortisol Secretion
Source: J Clin Endocrinol Metab. Author manuscript; Available in PMC 2026 Apr 7. (PMC13056330; doi:10.1210/clinem/dgaf515)
Supplement: Supplemental Tables [file NIHMS2153880-supplement-Supplemental_Tables.pdf]

Supplemental Table 1. Biochemical severity score (BSS) for hypercortisolism.

| Test* | Reference range | ACTH-dependent hypercortisolism | | ACTH-independent hypercortisolism | |
|---|---|---|---|---|---|
| | | Result | Points | Result | Points |
| **Post 1-mg DST serum cortisol** | <1.8 μg/dL | < 1.8 | 0 | < 1.8 | 0 |
| | | 1.8 - 3 | 1 | 1.8 - 3 | 1 |
| | | 3 - 5 | 2 | 3 - 5 | 2 |
| | | 5 - 10 | 3 | 5 - 10 | 3 |
| | | > 10 | 4 | > 10 | 4 |
| **24-hour urinary free cortisol** | 3.5 - 45 μg/24h | < 45 | 0 | < 45 | 0 |
| | | 45 - 100 | 2 | 45 - 100 | 2 |
| | | 101 - 200 | 3 | 101 - 200 | 3 |
| | | 201 - 300 | 4 | 201 - 300 | 4 |
| | | 300 - 500 | 5 | ≥ 301 | 5 |
| | | > 500 | 7 | > 500 | 7 |
| **Late night salivary cortisol** | < 50 ng/dL | 50 - 100 | 1 | 50 - 100 | 1 |
| | | 100 - 300 | 3 | 100 - 300 | 3 |
| | | > 300 | 4 | > 300 | 4 |
| **ACTH** | 10 - 60 pg/dL | - | - | > 15 | 0 |
| | | | | 6 - 15 | 1 |
| | | | | < 5 | 2 |
| **DHEA-S** | Sex- and age-dependent μg/dL | - | - | < 40 (if man or premenopausal woman)<br>< 25 (if postmenopausal woman) | 1 |
| | | | | < 15 (any sex and age) | 2 |

Biochemical scoring system[16] to grade severity was based on Endocrine Society guidelines on the diagnosis of Cushing syndrome[17], clinical guidelines on adrenal incidentaloma[18], systematic review on MACS[19], and expert opinion[16].

Abbreviations: ACTH, adrenocorticotropin hormone; DHEA-S, dehydroepiandrosterone sulfate; DST, dexamethasone suppression test

*The biochemical disease severity score was calculated prior to surgical treatment for hypercortisolism in each patient using their two most abnormal biochemical test results.

Mild biochemical severity score was defined as a sum of points between 0 and 3. Moderate biochemical severity score was defined as a sum of points between 4 and 6. Severe biochemical severity score was defined as a sum of points between 7 and 11.

Supplemental Table 2. Clinical severity score (CSS) for hypercortisolism.

<table>
<tr><th colspan="2">Clinical Characteristic</th><th>Points Assigned</th></tr>
<tr><td rowspan="6">Metabolic Abnormalities</td><td>Hypertension</td><td>1</td></tr>
<tr><td>Pre-diabetes or type 2 diabetes mellitus</td><td>1</td></tr>
<tr><td>Osteopenia</td><td>1</td></tr>
<tr><td>Osteoporosis by BMD and/or fragility fracture within the past 12 months</td><td>2</td></tr>
<tr><td>DVT/PE within the past 12 months</td><td>3</td></tr>
<tr><td>Weight gain</td><td>1</td></tr>
<tr><td rowspan="5">Physical Exam Features</td><td>Central obesity</td><td>1</td></tr>
<tr><td>Supraclavicular and/or dorsocervical fat accumulation</td><td>3</td></tr>
<tr><td>Rounding of the face with or without plethora</td><td>3</td></tr>
<tr><td>Skin changes (violaceous striae, thinning and/or bruising of the skin)</td><td>3</td></tr>
<tr><td>Proximal muscle weakness</td><td>4</td></tr>
<tr><td colspan="3">Clinical scoring system[16] to grade severity was based on Endocrine Society guidelines on the diagnosis of Cushing syndrome[17], clinical guidelines on adrenal incidentaloma[18], systematic review on MACS[19], and expert opinion[16].<br>Abbreviations: BMD, bone mineral density; DVT, deep venous thrombosis; PE, pulmonary embolism.<br>The clinical disease severity score was calculated for each patient prior to surgical treatment for hypercortisolism. Mild clinical severity score was defined as a sum of points between 1 and 8.<br>Moderate clinical severity score was defined as a sum of points between 9 and 14. Severe clinical severity score was defined as a sum of points between 15 and 22.</td></tr>
</table>

Supplemental Table 3. Subgroup analysis of adrenal insufficiency prior to and after 2017.

| | Overall | Year of adrenalectomy | | P value |
|---|---|---|---|---|
| | | ≤2017 | >2017 | |
| Adrenal Insufficiency | 153 (55%) | 62/108 (57%) | 91/173 (52%) | NS |
| Basal cortisol <10 μg/dL | 169 (60%) | 66/106 (61%) | 103/173 (60%) | NS |
| CST cortisol < 18 μg/dL | 162 (58%) | 65/108 (60%) | 97/173 (56%) | NS |

Abbreviations: CST, cosyntropin stimulation test.

Supplemental Table 4. Characteristics of patients <60 years ≥60 years.

| **Variable** | **Age <60 n=171 (61%)** | **Age ≥60 n=110 (39%)** | ***P* value** |
|---|---|---|---|
| Women, n (%) | 149 (87%) | 77 (70%) | 0.0004* |
| BMI, median (IQR), kg/m$^2$ | 33.4 (27.7-39.5) | 31.4 (25.9-36.2) | 0.05 |
| Mode of discovery, n (%)<br>Incidental | 144 (84.2%) | 89 (80.9%) | 0.47 |
| Laterality<br>Unilateral<br>Bilateral | 137 (80%)<br>34 (20%) | 82 (75%)<br>28 (25%) | 0.27 |
| Time from adenoma diagnosis to adrenalectomy in months, median (IQR) | 15.5 (4.6-58.6) | 11.7 (5.7-31.3) | 0.37 |
| Resected adenoma size in cm, median (IQR) | 3.1 (2.3-3.9) | 3.0 (2.3-3.7) | 0.38 |
| Resected adenoma density in HU, median (IQR) | 12 (0-25) | 9 (0-19) | 0.11 |
| ACTH in pg/dL, median (IQR) | 6.2 (5-12) | 8.6 (5-14) | 0.02* |
| DHEA-S in µg/dL, median (IQR) | 40.4 (21.1-72) | 35.6 (18-58.5) | 0.08 |
| 1-mg DST cortisol in µg/dL, median (IQR) | 3.5 (2.6-7.3) | 3.0 (2.5-4.7) | 0.04* |
| Biochemical severity score, mean (SD) | 4.1 (1.9) | 3.8 (1.5) | 0.17 |
| Clinical severity score, mean (SD) | 7.2 (5.2) | 6.4 (4.8) | 0.46 |
| Adrenal insufficiency, n (%) | 110 (64.3%) | 43 (39.1%) | <.0001* |
| Basal cortisol <10 µg/dL, n (%) | 116 (67.8%) | 53 (48.1%) | 0.001* |
| CST 60-minute cortisol <18 µg/dL, n (%) | 116 (67.8%) | 46 (41.8%) | <.0001* |
| Concordant basal and CST cortisol, n (%) | 139 (81.3%) | 79 (71.8%) | 0.06 |
| Abbreviations used: ACTH, adrenocorticotropin hormone; BMI, body mass index, CST, cosyntropin stimulation test; DHEA-S, dehydroepiandrosterone sulfate; DST, dexamethasone suppression test; IQR, interquartile range | | | |

Supplemental Table 5. Univariable and multivariable regression analysis models of adrenal insufficiency risk factors.

| Variable | Univariable Analysis | | Multivariable Analysis | | | | | |
|---|---|---|---|---|---|---|---|---|
| | | | Model 1: Adjustment for Biochemical Severity Score and additional factors[§] | | Model 2: Adjustment for Clinical Severity Score and additional factors[¶] | | Model 3: Adjustment for Adenoma Size and additional Factors[¥] | |
| | Odds Ratio (95% CI) | *P* value | Odds Ratio (95% CI) | *P* value | Odds Ratio (95% CI) | *P* value | Odds Ratio (95% CI) | *P* value |
| Age (per 10 years) | 0.08 (0.02-0.30) | <.0001* | 0.67 (0.53-0.84) | <.001* | 0.66 (0.52-0.83) | <.001* | 0.65 (0.51-0.82) | <.001* |
| Sex<br>Male<br>Female | Reference<br>1.88 (1.04-3.45) | 0.03* | Reference<br>1.52 (0.81-2.86) | 0.18 | Reference<br>1.62 (0.86-3.08) | 0.13 | Reference<br>1.62 (0.86-3.08) | 0.13 |
| BMI (per 1 unit) | 0.99 (0.96-1.02) | 0.63 | 0.97 (0.94-1.01) | 0.20 | 0.98 (0.94-1.01) | 0.28 | 0.97 (0.93-1.00) | 0.09 |
| Laterality<br>Unilateral<br>Bilateral | Reference<br>0.56 (0.32-1.00) | 0.05 | Reference<br>0.58 (0.32-1.06) | 0.08 | Reference<br>0.63 (0.34-1.15) | 0.13 | Reference<br>0.54 (0.29-1.00) | 0.05 |
| Tumor size (per 1 cm) | 1.12 (0.94-1.35) | 0.18 | -- | | -- | | 1.25 (1.03-1.53) | 0.02* |
| Biochemical Severity Score | 1.14 (1.01-1.3) | 0.04* | 1.14 (0.99-1.31) | 0.06 | -- | | -- | |
| Clinical Severity Score | 0.91 (0.37-2.20) | 0.83 | -- | | 0.99 (0.94-1.05) | 0.89 | -- | |
| Abbreviations: BMI, body mass index | | | | | | | | |

[§]The model was adjusted for age, sex, BMI, adenoma laterality, and Biochemical Severity Score.

[¶]The model was adjusted for age, sex, BMI, adenoma laterality, and Clinical Severity Score.

[¥] The model was adjusted for age, sex, BMI, adenoma laterality, and adenoma size.

Supplemental Table 6. Univariable and multivariable regression analysis models of risk factors for basal cortisol <10 µg/dL.

| **Variable** | **Univariable Analysis** | | **Multivariable Analysis with Adjustment for Biochemical Severity Score and Additional Factors**[§] | | **Multivariable Analysis with Adjustment for Clinical Severity Score and Additional Factors**[¶] | | **Multivariable Analysis with Adjustment for Adenoma Size and Additional Factors**[¥] | |
|---|---|---|---|---|---|---|---|---|
| | Odds Ratio (95% CI) | *P* value | Odds Ratio (95% CI) | *P* value | Odds Ratio (95% CI) | *P* value | Odds Ratio (95% CI) | *P* value |
| Age (per 10 years) | 0.65 (0.51-0.81) | <0.001* | 0.64 (0.50-0.81) | <.001* | 0.64 (0.50-0.81) | <.001* | 0.62 (0.49-0.79) | <.001* |
| Sex<br>Male<br>Female | <br>Reference<br>1.59 (0.88-2.90) | 0.12 | 1.25 (0.66-2.33) | 0.47 | <br>Reference<br>1.36 (0.72-2.56) | 0.33 | <br>Reference<br>1.34 (0.71-2.53) | 0.35 |
| BMI (per 1 unit) | 0.98 (0.95-1.01) | 0.36 | 0.97 (0.94-1.00) | 0.09 | 0.97 (0.94-1.00) | 0.14 | 0.96 (0.93-0.99) | 0.02* |
| Laterality<br>Unilateral<br>Bilateral | <br>Reference<br>0.75 (0.42-1.34) | 0.33 | 1.25 (0.66-2.33) | 0.41 | <br>Reference<br>0.86 (0.47-1.57) | 0.62 | <br>Reference<br>0.72 (0.38-1.34) | 0.30 |
| Tumor size (per 1 cm) | 1.19 (0.99-1.45) | 0.06 | -- | | -- | | 1.32 (1.08-1.64) | 0.01* |
| Biochemical Severity Score | 1.17 (1.02-1.35) | 0.02* | 1.17 (1.01-1.36) | 0.03* | -- | | -- | |
| Clinical Severity Score | 0.99 (0.94-1.03) | 0.71 | -- | | 0.99 (0.94-1.04) | 0.83 | -- | |
| Abbreviations: BMI, body mass index | | | | | | | | |

[§]The model was adjusted for age, sex, BMI, adenoma laterality, and Biochemical Severity Score.

[¶]The model was adjusted for age, sex, BMI, adenoma laterality, and Clinical Severity Score.

[¥] The model was adjusted for age, sex, BMI, adenoma laterality, and adenoma size.

Supplemental Table 7. Univariable and multivariable regression analysis models for 60-minute cortisol <18 µg/dL.

| **Variable** | **Univariable Analysis** | | **Multivariable Analysis with Adjustment for Biochemical Severity Score and Additional Factors**[§] | | **Multivariable Analysis with Adjustment for Clinical Severity Score and Additional Factors**[¶] | | **Multivariable Analysis with Adjustment for Adenoma Size and Additional Factors**[¥] | |
|---|---|---|---|---|---|---|---|---|
| | Odds Ratio (95% CI) | P value | Odds Ratio (95% CI) | P value | Odds Ratio (95% CI) | P value | Odds Ratio (95% CI) | P value |
| Age (per 10 years) | 0.63 (0.50-0.78) | <.0001* | 0.64 (0.50-0.82) | 0.0005* | 0.64 (0.50-0.81) | 0.0004* | 0.61 (0.47-0.77) | <.001* |
| Sex<br>Male<br>Female | <br>Reference<br>1.53 (0.85-2.78) | 0.15 | <br><br>1.19 (0.62-2.27) | 0.59 | <br>Reference<br>1.32 (0.68-2.54) | 0.39 | <br>Reference<br>1.35 (0.68-2.68) | 0.37 |
| BMI (per 1 unit) | 0.99 (0.96-1.02)) | 0.63 | 0.97 (0.94-1.00) | 0.12 | 0.97 (0.94-1.01) | 0.21 | 0.95 (0.92-0.99) | 0.01* |
| Laterality<br>Unilateral<br>Bilateral | <br>Reference<br>0.25 (0.13-0.46) | <.0001* | <br>Reference<br>0.24 (0.12-0.44) | <.0001* | <br>Reference<br>0.27 (0.14-0.50) | <.0001* | <br>Reference<br>0.17 (0.08-0.35) | <.0001* |
| Tumor size (per 1 cm) | 1.29 (1.07-1.57) | 0.009* | -- | -- | -- | | 1.64 (1.31-2.10) | <.0001* |
| Biochemical Severity Score | 1.16 (1.02-1.33) | 0.02* | 1.22 (1.05-1.42) | 0.01* | -- | | -- | |
| Clinical Severity Score | 0.98 (0.93-1.02) | 0.43 | -- | -- | 0.99 (0.94-1.05) | 0.85 | -- | |
| Abbreviations: BMI, body mass index | | | | | | | | |

[§]The model was adjusted for age, sex, BMI, adenoma laterality, and Biochemical Severity Score.

[¶]The model was adjusted for age, sex, BMI, adenoma laterality, and Clinical Severity Score.

[¥] The model was adjusted for age, sex, BMI, adenoma laterality, and adenoma size.

Supplemental Table 8. Adrenal insufficiency recovery based on biochemical and clinical severity.

**Biochemical Severity Groups**

| Group | Median Time | Lower 95% | Upper 95% | 25% Failures | 75% Failures |
|---|---|---|---|---|---|
| Mild | 3.0226 | 2.3984 | 3.9097 | 1.5113 | 7.6552 |
| Moderate | 4.7311 | 2.7927 | 7.1623 | 1.9384 | 13.799 |
| Severe | 14.489 | 5.7167 | 21.881 | 5.7167 | 21.881 |
| Combined | 3.9097 | 3.0226 | 5.9796 | 1.9384 | 14.128 |

| Test | ChiSquare | DF | Prob>ChiSq |
|---|---|---|---|
| Log-Rank | 7.2628 | 2 | 0.0265* |
| Wilcoxon | 10.2325 | 2 | 0.0060* |

**Clinical Severity Groups**

| Group | Median Time | Lower 95% | Upper 95% | 25% Failures | 75% Failures |
|---|---|---|---|---|---|
| Mild | 3.3183 | 2.8255 | 5.2896 | 1.7084 | 9.0022 |
| Moderate | 3.9097 | 2.267 | 13.799 | 1.9384 | 14.489 |
| Severe | 11.926 | 4.534 | . | 6.5052 | . |
| Combined | 3.9097 | 3.0226 | 5.9796 | 1.9384 | 14.128 |

| Test | ChiSquare | DF | Prob>ChiSq |
|---|---|---|---|
| Log-Rank | 6.1247 | 2 | 0.0468* |
| Wilcoxon | 6.2611 | 2 | 0.0437* |
